# Transcriptional Dynamics and Key Regulators of Adipogenesis in Mouse Embryonic Stem Cells: Insights from Robust Rank Aggregation Analysis

**DOI:** 10.3390/ijms25179154

**Published:** 2024-08-23

**Authors:** Mouza Alzaabi, Mariam Khalili, Mehar Sultana, Mohamed Al-Sayegh

**Affiliations:** 1 Division of Biology, New York University Abu Dhabi, Saadiyaat Island, Abu Dhabi P.O. Box 129188, United Arab Emirates; maa9608@nyu.edu (M.A.); mk8962@nyu.edu (M.K.); 2 Center for Genomics & Systems Biology, New York University Abu Dhabi, Saadiyaat Island, Abu Dhabi P.O. Box 129188, United Arab Emirates; ms8783@nyu.edu

**Keywords:** adipogenesis, embryonic stem cells, transcriptomics, robust ranking aggregation

## Abstract

Embryonic stem cells are crucial for studying developmental biology due to their self-renewal and pluripotency capabilities. This research investigates the differentiation of mouse ESCs into adipocytes, offering insights into obesity and metabolic disorders. Using a monolayer differentiation approach over 30 days, lipid accumulation and adipogenic markers, such as *Cebpb*, *Pparg*, and *Fabp4,* confirmed successful differentiation. RNA sequencing revealed extensive transcriptional changes, with over 15,000 differentially expressed genes linked to transcription regulation, cell cycle, and DNA repair. This study utilized Robust Rank Aggregation to identify critical regulatory genes like PPARG, CEBPA, and EP300. Network analysis further highlighted *Atf5*, *Ccnd1*, and *Nr4a1* as potential key players in adipogenesis and its mature state, validated through RT-PCR. While key adipogenic factors showed plateaued expression levels, suggesting early differentiation events, this study underscores the value of ESCs in modeling adipogenesis. These findings contribute to our understanding of adipocyte differentiation and have significant implications for therapeutic strategies targeting metabolic diseases.

## 1. Introduction

Embryonic stem cells (ESCs) possess remarkable self-renewal and pluripotency capabilities, making them invaluable tools for developmental biology and regenerative medicine [1]. ESCs can differentiate into various cell types, offering insights into lineage commitment and the molecular underpinnings of development. One crucial differentiation pathway is adipogenesis, the process through which preadipocytes mature into adipocytes, which is essential for understanding obesity, metabolic disorders, and potential therapeutic avenues for adipose-tissue-related conditions. Adipogenesis has been extensively studied in various cell models, including preadipocyte cell lines (e.g., 3T3-L1) [2], mesenchymal stem cells (MSCs) [3], and embryonic fibroblasts [4,5]. However, using ESCs to study adipogenesis offers distinct advantages. ESCs provide a more physiologically relevant model for early development and differentiation processes, enabling researchers to investigate the initial stages of adipocyte lineage commitment and the factors influencing this process from a pluripotent state [6]. This can lead to a deeper understanding of the molecular mechanisms underlying adipogenesis and the potential discovery of novel regulatory pathways [6].

Mouse ESCs, derived from the inner cell mass of blastocysts, have the unique ability to proliferate indefinitely while maintaining the potential to differentiate into all three germ layers [1,7,8]. This pluripotency is regulated by a network of transcription factors, including Oct4, Sox2, and Nanog, which maintain the undifferentiated state and prevent lineage-specific gene expression [9,10]. The differentiation of ESCs into specific cell types involves complex signaling pathways and epigenetic modifications, making them an ideal model for studying early developmental processes [11,12]. Recent advancements in RNA sequencing have further elucidated the heterogeneity and plasticity within ESC populations, highlighting subpopulations with distinct differentiation potentials. These findings underscore the dynamic nature of ESCs and the intricate regulatory networks governing their pluripotency and differentiation [13]. ESC has been utilized in various studies due to its capability to differentiate in vitro into various generic specialized cell types, including neurons [14], blood vessels [15], hepatocytes [16], and osteocytes [17]. In addition to cell types, several studies have utilized ESC differentiation to understand adipogenesis [6,18,19,20].

Adipogenesis is a highly regulated process that plays a critical role in energy homeostasis and metabolism. It involves the conversion of mesenchymal stem cells or preadipocytes into mature adipocytes characterized by lipid accumulation and the expression of adipogenic markers, such as PPARγ and C/EBPα [21,22]. Key signaling pathways involved in adipogenesis include the Wnt/β-catenin pathway, the insulin signaling pathway, and the BMP signaling pathway, all of which contribute to the regulation of adipocyte differentiation and function [22,23,24]. Recent studies have identified additional molecular regulators of adipogenesis, such as miRNAs and long non-coding RNAs (lncRNAs), which modulate gene expression and cellular function during adipocyte differentiation [25,26]. Dysregulation of adipogenesis is associated with metabolic disorders, including obesity, insulin resistance, and type 2 diabetes [22,27,28]. Therefore, understanding the molecular mechanisms governing adipogenesis is of great importance for developing therapeutic strategies to combat these conditions. Given the complexities of these metabolic conditions as well as the dynamical nature of adipogenesis, global genome, specifically transcriptional, changes can occur. Therefore, investigating the transcriptomic changes during the differentiation of ESCs into adipocytes offers valuable insights into the fundamental processes of cell fate determination and tissue development. From a clinical perspective, this knowledge can inform the development of regenerative therapies and interventions for metabolic diseases. By elucidating the key regulatory networks and signaling pathways involved in adipogenesis, researchers can identify potential targets for therapeutic intervention and enhance our understanding of adipose tissue biology [22,29,30]. For instance, the advent of CRISPR/Cas9 genome editing technology has revolutionized functional genomics, allowing for the precise manipulation of genes involved in adipogenesis. This technology enables the identification and validation of novel adipogenic regulators, providing deeper insights into the genetic and epigenetic control of adipocyte differentiation [31,32]. However, current approaches address computational/algorithmic analysis within gene expression changes, such robust ranking aggregation (RRA) [33].

This study employs RRA to analyze the transcriptomic data obtained from mouse ESCs undergoing adipogenesis. RRA is a powerful statistical method designed to identify differentially expressed genes with high confidence, even in the presence of noise and variability [33]. By ranking genes based on their expression changes across multiple conditions, RRA provides a robust framework for identifying key regulatory elements and pathways involved in adipocyte differentiation. This approach is particularly beneficial for understanding the dynamic and complex nature of gene expression during cellular differentiation [34]. RRA has been successfully applied in various fields of biomedical research, demonstrating its utility in integrating heterogeneous datasets and identifying robust biomarkers and therapeutic targets [35,36]. By leveraging the power of RRA, this study aims to provide a comprehensive analysis of the transcriptomic changes during ESC-to-adipocyte differentiation, revealing either critical regulatory genes and/or novel insights. This knowledge can have significant implications for both basic biology and clinical applications, offering potential avenues for therapeutic interventions in metabolic disorders and advancing our understanding of adipose tissue development and fat differentiation.

## 2. Results

### 2.1. Capacity of Mouse ESC (mESC::HB9::GFP) to Differentiate into Adipogenic-like Features

**Figure 1:**
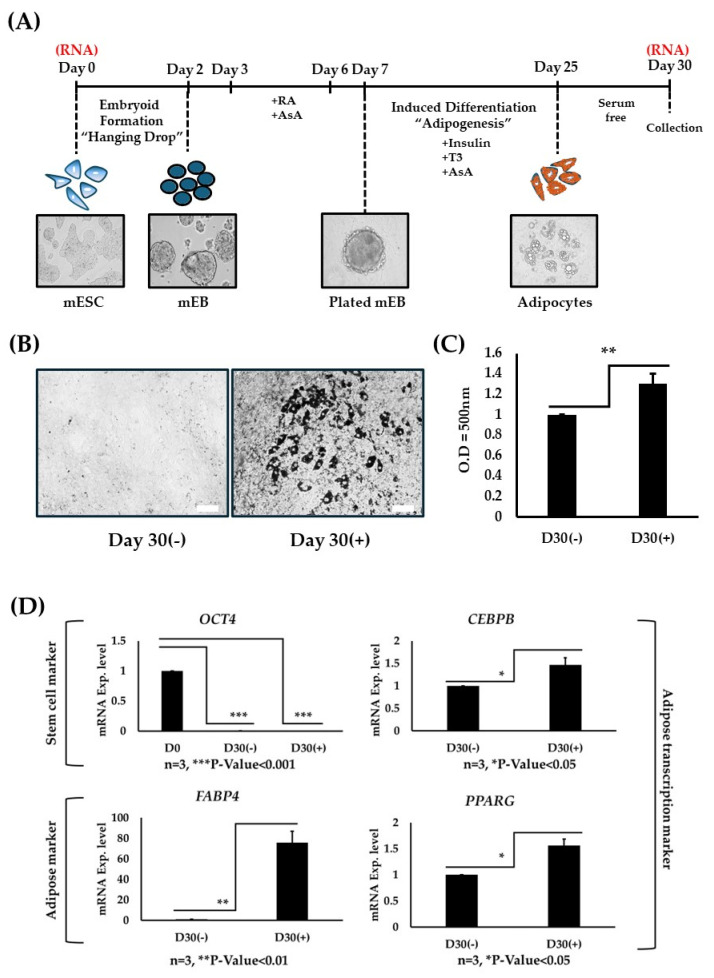
(**A**) Schematic experimental workflow of mouse ESC undergoing adipogenic differentiation with specified phase timepoints. Relevant brightfield images are presented at the indicated timepoints. RNA (in red) was collected at the indicated timepoint. (**B**) Oil Red O staining in non-differentiating (day 30(−)) and differentiating (day 30(+)) conditions; white bar scales to 100 um. (**C**) Spectrometry of lipid triglyceride particle isolates formed by staining at an optical density (O.D) of 500 nm; values are normalized to D30(−) (n = 3; ** *p*-Value < 0.01). (**D**) RT-PCR quantification of the relevant biomarkers, including *Oct4*, *Cebpb*, *Pparg*, and *Fabp4*. Apart from *Oct4*, which was normalized to the beginning timepoint (D30(−)/D0 and D30(+)/D0), the remaining genes were normalized based on the condition (D30(+)/D30(−)); all genes were run in triplicates, as indicated.

To comprehend the capability of ESC to differentiate into adipogenic-like features, the mESC::*hb9*::GFP cell line, a well-established motor neuron progenitor cell line [37], was used in this study. Based on previous studies [19,20], a monolayer experimental layout designed was performed, encompassing a period of 25 days with or without adipogenic differentiating factors followed by 5 days with serum-free medium (Figure 1A). On day 30, non-differentiating media did not show signs of lipid formation, whereas in the differentiation media condition there was more lipid formation present, as evidenced by O Red Oil staining (Figure 1B). Furthermore, quantification of lipid particles was observed to be statistically significant (Figure 1C). To make in-depth observations at the molecular level, specific gene markers were tested, including pluripotent stem cell marker *Oct4* [19], adipogenic transcription markers *Cebpb* and *Pparg*, and mature adipose marker *Fabp4* [4,5]. The pluripotent stem cell marker *Oct4* was significantly downregulated at day 30 under differentiating and non-differentiating conditions as opposed to day 0 (Figure 1D). Meanwhile, when comparing the adipogenic markers at day 30 (differentiation/non-differentiation), there was a slight increase in the transcriptional markers *Cebpb* and *Pparg* and a significant increase in the mature adipose tissue marker *Fabp4*. Collectively, these observations suggest that adipogenesis progressed throughout this experimental layout.

### 2.2. RNA-Seq Correlation Analysis Displays Global Differential Gene Expression between Differentiating and Non-Differentiating Conditions

To obtain insight into the global transcriptome profile changes between conditions at the beginning timepoint of day 0 and the end of the experiment at day 30, RNA-Seq was performed on RNA samples from day 0 (D0), day 30 non-differentiating condition (D30(−) or D30min), and day 30 differentiating condition (D30(+) or D30 pos).

**Figure 2:**
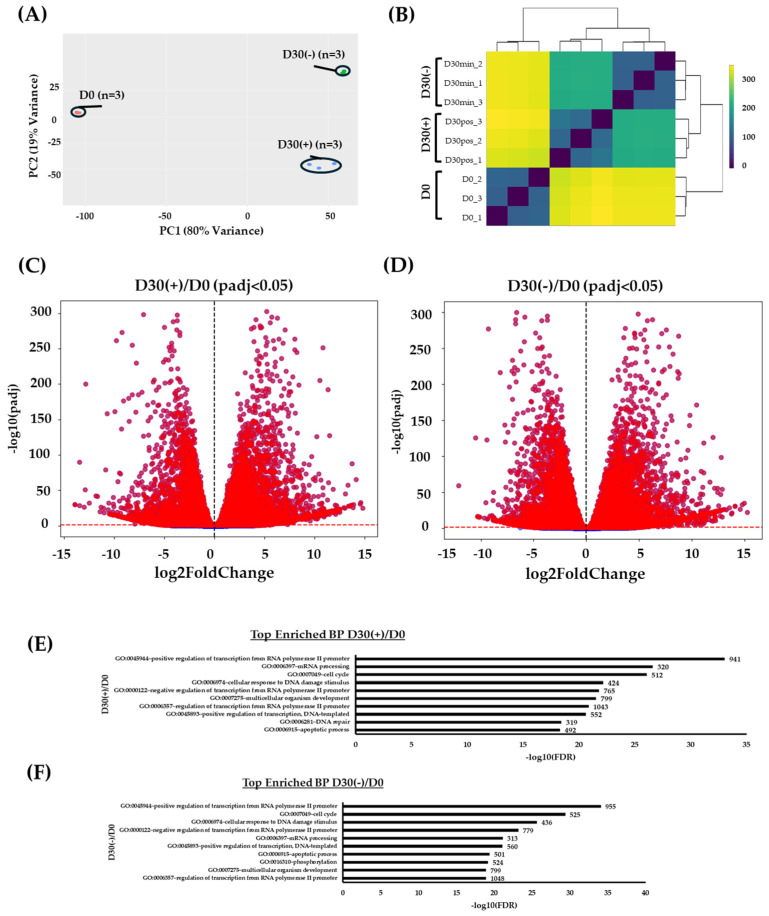
(**A**) Principal component analysis (PCA) of conditions D0, D30(−), and D30(+) with tight clustering of three biological replicates displaying the highest variance at 80% (PC1) followed by 19% (PC2). (**B**) Metric distance heatmap displaying the variance as observed in the previous PCA. (**C**) Volcano plot representation of DEGs in D30(+)/D0 and (**D**) D30(−)/D0 (*y*-axis: normalized statistical significance at −log10(P-adj); *x*-axis: normalized fold change of gene expression at log 2(Fold Change); the vertical line represents the cut-off of P-adj < 0.05, whereas the horizontal line represents the fold change at 0). (**E**) GoTerm enrichment analysis of top 10 biological processes in D30(+)/D0 and (**F**) D30(−)/D0. Enrichments are based on false dependent rate (FDR) values normalized with −log10(FDR).

The correlation analysis displayed variable changes between all three conditions (Figure 2A,B). As illustrated in the principal component analysis, the main factor of this was driven by time, followed by the conditions, with PC1 at 80% and PC2 at 19% (Figure 2A). This has also been reaffirmed accordingly in the distance heatmap (Figure 2B). Due to this, differentially expressed genes (DEGs) were investigated mainly according to the condition over time (i.e., D30(+)/D0 and D30(−)/D0).

To isolate relevant DEGs, a statistical cut-off of P-adj < 0.05 was set for both D30(−)/D0 and D30(+)/D0 (Figure 2C,D). Based on this cut-off, there were 15,180 and 15,016 DEGs identified in D30(−)/D0 and D30(+)/D0, respectively. These genes were further investigated via gene ontology enrichment analysis, specifically at the biological process’s levels (Figure 2E,F; Supplementary File: Tables S1 and S2). In the differentiation condition of D30(+)/D0, there were several processes that are involved in molecular and transcription regulation as well as DNA damage response and the cell cycle (GO:0045944~positive regulation of transcription from RNA polymerase II promoter, GO:0007049~cell cycle, GO:0006281~DNA repair) (Figure 2E; Table S1). Likewise, these terms were also observed in the non-differentiation condition of D30(−)/D0 (Figure 2F; Table S2). Therefore, this suggests that the highest impact of change is focused on transcriptional mechanisms, which may display changes in gene expression between the conditions.

### 2.3. Isolation of Adipose Tissue, Lipid, and/or Fat-Related DEG Genes and Their Expression

**Figure 3:**
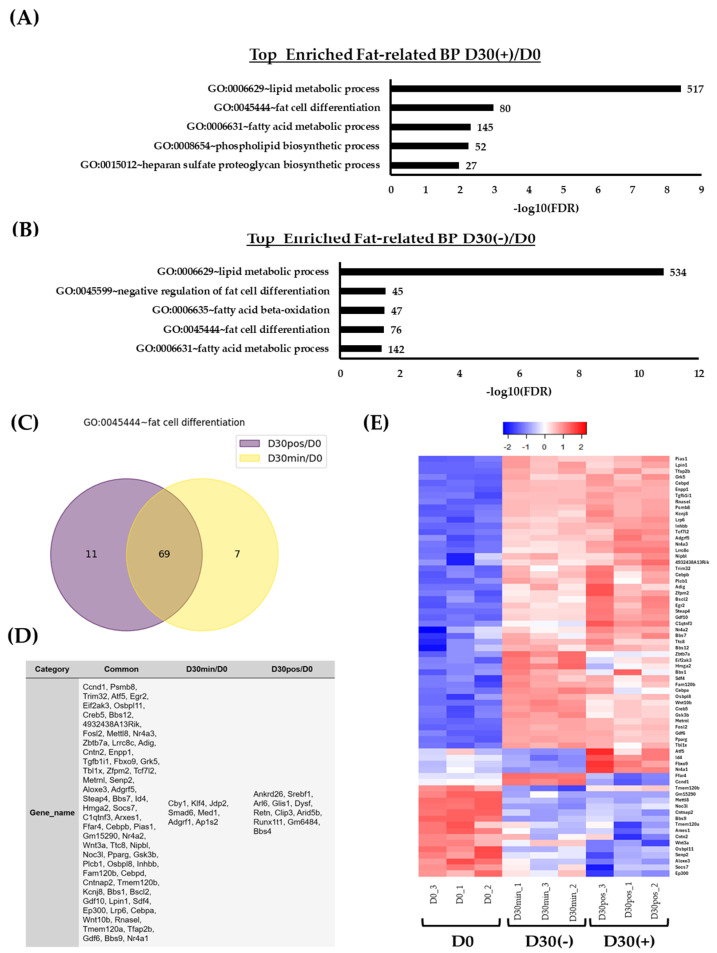
(**A**) GoTerm enrichment analysis of top 5 biological processes in (+)/D0 and (**B**) D30(−)/D0 filtered for “adipose”, “lipid”, and/or “fat” terms. Enrichments are based on the false dependent rate (FDR) values normalized with −log10(FDR). (**C**) Venn diagram comparing DEGs of the term GO:0045444~fat cell differentiation displaying common and unique genes in D30(−)/D0 and (+)/D0. (**D**) List of DEGs that are common between D30(−)/D0 and (+)/D0 or uniquely expressed in either D30(−)/D0 or (+)/D0. (**E**) A metric heatmap displaying expression patterns of each replicate in conditions D0, D30(−), and (+) of the common DEG genes identified in GO:0045444~fat cell differentiation. The heatmap is scaled to a log2 count per million [log2cpm] and a Z-Score (blue = downregulation, red = upregulation).

To specify the previous DEG list into specific terms related to regulatory features of adipogenesis, terms were further filtered into “adipose tissue”, “lipids”, and/or “fat” (Figure 3). In (+)/D30 and D30(−)/D0 conditions, there were several terms related to the features of adipose tissue; however, only with the statistically significant enrichments were GoTerms further investigated. Commonly, in both conditions, these included lipid metabolic process, fat cell differentiation, and the fatty acid metabolic process (Figure 3A,B; Tables S3 and S4).

More specifically, the top 5 (among 36 enriched terms) enriched terms in (+)/D30 DEGs included GO:0006629~lipid metabolic process (517 genes), GO:0045444~fat cell differentiation (80 genes), GO:0006631~fatty acid metabolic process (145 genes), GO:0008654~phospholipid biosynthetic process (52 genes), and GO:0015012~heparan sulfate proteoglycan biosynthetic process (27 genes) (Figure 3A; 3S). However, the list included GO:0050873~brown fat cell differentiation (35 genes), GO:0019915~lipid storage (27 genes), GO:0045600~positive regulation of fat cell differentiation (42 genes), GO:0050872~white fat cell differentiation (21 genes), and GO:0060612~adipose tissue development (38 genes) (Table S3).

In D30(−)/D0, the top 5 (among 31 enriched terms) included GO:0006629~lipid metabolic process (534 genes), GO:0045599~negative regulation of fat cell differentiation (45 genes), GO:0006635~fatty acid beta-oxidation (47 genes), GO:0045444~fat cell differentiation (76 genes), and GO:0006631~fatty acid metabolic process (142 genes) (Figure 3B). However, the list included GO:0045165~cell fate commitment (60 genes), GO:0048665~neuron fate specification (22 genes), GO:0045600~positive regulation of fat cell differentiation (41 genes), GO:0060612~adipose tissue development (38 genes), and GO:0090336~positive regulation of brown fat cell differentiation (15 genes) (Table S4).

Even though there are variable enrichment terms between both conditions, DEGs in GO:0045444~fat cell differentiation were further investigated to delineate a common list (Figure 3C,D). There were 69 common genes that were differentially expressed between (+)/D0 and D30(−)/D0 (Figure 3C,D). Meanwhile, 11 and 7 genes were shown to be uniquely differentially expressed in (+)/D0 and D30(−)/D0, respectively (Figure 3C,D). The unique genes in (+)/D0 included *Ankrd26*, *Srebf1*, *Arl6*, *Glis1*, *Dysf*, *Retn*, *Clip3*, *Arid5b*, *Runx1t1*, *Gm6484* (*Angptl8*), and *Bns4* (Figure 3D), whereas in D30(−)/D0 the unique genes included *Cby1*, *Klf4*, *Jdp2*, *Smad6*, *Med1*, *Adgrf1*, and *Ap1s2* (Figure 3D).

The expressions of common DEGs shared between (+)/D0 and D30(−)/D0 were further investigated (Figure 3E). The heatmap revealed variable expression-pattern levels among conditions based on the scaled level. For instance, there were exclusive upregulation expressions of genes in the (+) condition, which included *Atf5*, *Id4*, *Fbxo9*, and *Nr4a1*. As opposed to this, there were exclusive upregulation expressions in D30(−), such as *Ffar4* and *Ccnd1*. In addition to these observations, there were several genes that were observed to be downregulated in (+), including *Ep300*, *Socs7*, *Aloxe3*, *Senp2*, *Osbpl11*, and *Wnt3a*. The remaining genes were observed to be differentially upregulated in both (+) and D30(−) as opposed to D0.

### 2.4. Network Analysis of Common DEGs and RRA

**Figure 4:**
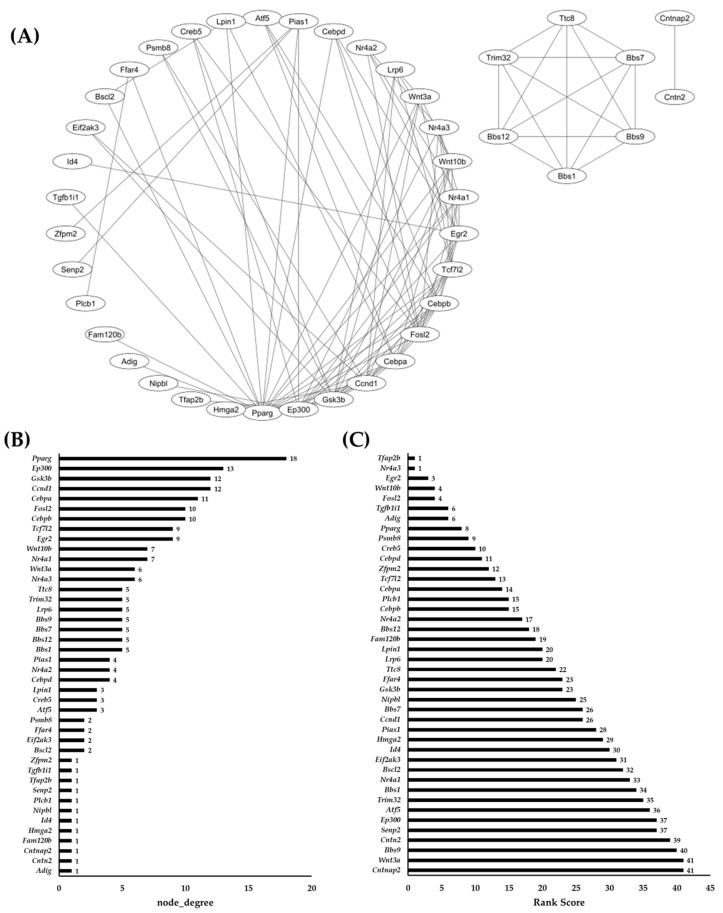
(**A**) A PPI interaction network (based on STRING and modified via Cytoscape) displaying interactions among the protein products of common DEGs identified previously. (**B**) Chart showing centrality by node degrees, where the higher the node the more central it is to the whole network. (**C**) Robust Rank Aggregation (RRA) score chart demonstrating the significant expression of DEGs in the (+)/D0 and D30(−)/D0 dataset. The lower the score, the higher the biological significance and effect.

To further decipher the identified common DEGs of (+)/D0 and D30(−)/D0, a protein-to-protein interaction (PPI) was constructed (Figure 4A). The aim of this construct was to identify the centrality between the protein products of DEGs and therefore gain insight into their biological and/or molecular roles. Based on this, the construct identified 43 proteins that are interconnected with other proteins (Figure 4B). PPARG showed the highest centrality by having 18 node degrees, whereas CEBPA and CEBPB were ranked fourth (11 node degrees) and fifth (10 node degrees), respectively (Figure 4B). EP300 was ranked second with 14 node degrees, whereas GSK3B and CCND1 were ranked third with 12 node degrees. Interestingly, FFAR4 was ranked twelfth with two node degrees, whereas ATF5 was ranked eleventh (three node degrees) and NR4A1 was ranked seventh (seven node degrees), along with WNT10B. Lastly, ID4 was observed to be ranked 13 with a node degree of one.

To further identify and rank these DEGs based on changes in expression across conditions (+)/D0 and D30(−)/D0 data, RRA was applied after eliminating all proteins that had a node degree less than one (Figure 4C). The lower RRA score indicates that a gene consistently appears at top rank lists across the datasets, whereas a higher RRA score indicates that a gene less consistently appears to the top rank across the datasets (Figure 4C). The top-ranked genes identified were *Cntnap2* and *Wnt3a* with a shared score of 41. This is followed by *Bbs9* (score = 40), *Cntn2* (score = 39) and *Senp2* (score = 37). Interestingly, *Ep300* was also observed at a score of 37, followed by *Atf5* at a score of 36. For the remaining genes with variable expression patterns observed previously, *Nr4a1* scored 33, *Id4* scored 30, *Ccnd1* scored 26, and *Ffar4* scored 23. As for the known adipogenic markers, *Cebpb* was observed to have a score of 15, *Cebpa* scored 14, *Cebpd* scored 11, and *Pparg* scored 8.

### 2.5. Expression and RT-PCT Validation of Atf5, Ccnd1, and Nr4a1:

**Figure 5:**
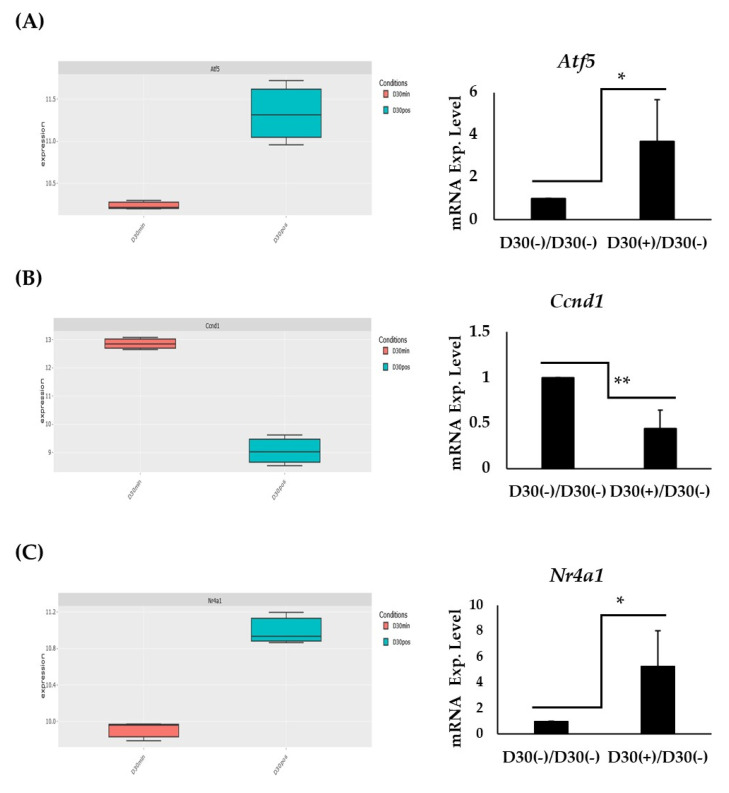
(**A**) Left panel: boxplot *Atf5* expression based on the RNA-Seq data. Right panel: RT-PCR of gene *Atf5* displaying mRNA expression levels (n = 3; * *p*-Value < 0.05). (**B**) Left panel: boxplot *Ccnd1* expression based on the RNA-Seq data. Right panel: RT-PCR of gene *Ccnd1* displaying mRNA expression levels (n = 3; ** *p*-Value < 0.01). (**C**) Left panel: boxplot *Nr4a1* expression based on the RNA-Seq data. Right panel: RT-PCR of gene *Nr4a1* displaying mRNA expression levels (n = 3; * *p*-Value < 0.05).

To validate some of the genes that showed interesting expression patterns, RT-PCR was utilized to validate *Atf5, Ccnd1,* and *Nr4a1* (Figure 5). Firstly, in silico data were visualized of each gene, which corresponded to the expression observed in the heatmap. It showed upregulation of genes *Atf5* (Figure 5A—left panel) and *Nr4a1* (Figure 5C—left panel) in (+) as opposed to D30(−). Conversely, *Ccnd1* was observed to be downregulated in (+) as opposed to D30(−) (Figure 5B—left panel). RT-PCR showed significant upregulation of *Atf5* (Figure 5A—right panel) and *Nr4a1* (Figure 5C—right panel) in (+) as opposed to D30(−). Meanwhile, RT-PCR showed significant downregulation of *Ccnd1* in (+) as opposed to D30(−) (Figure 5B—right panel). Collectively, RT-PCR results corresponded well with the datasets.

## 3. Discussion

ESCs offer a unique platform to investigate adipogenesis, providing insights into early developmental processes and regulatory mechanisms governing cell fate determination [22]. In this study, the adipogenic dynamical potential of mouse ESCs was explored using the computational approach of RRA. Applying this approach identified several key genes that would be characterized features of adipogenesis and/or mature adipose tissue.

The protocol approached in this study uses previous studies [6,18,19,20] that utilize ESC to understand adipogenesis. Although there are several approaches, the monolayer-directed differentiation was employed with the potential of collecting secreted material from formed adipocytes. This involved the exposure of attached EBs with soluble differentiating factors for a fixed period followed by incubation under low- to no-growth medium, such as serum-free medium. However, the main question at hand would be whether this reverses adipocyte differentiation or halts it, which remains unknown. Yet our findings demonstrate that under this approach, the mouse ESCs exhibited significant adipogenic-like features. This was characterized by lipid accumulation and upregulation of known adipogenic markers, including *Cebpb, Pparg,* and *Fabp4* in the differentiating condition as opposed to the non-differentiating condition. Indeed, the expression of this gene might be indicative of cell specification, especially as *Oct4*, one of the stem cell markers, was observed to decrease in both differentiating and non-differentiating conditions. Therefore, these observations underscore the robustness of ESCs as a model system for studying adipocyte differentiation from a pluripotent state. Comparative analysis with existing adipogenesis models, such as mesenchymal stem cells (MSCs) and preadipocyte cell lines, reveals distinct advantages of using ESCs. ESCs offer a more physiologically relevant model for early adipogenic events, enabling the study of initial lineage commitment and regulatory mechanisms not fully accessible in other systems.

Due to the dynamical transcriptional nature of adipogenesis at global genomic level, it was necessary to observe profile changes across the conditions. Bulk-RNA-Seq analysis in this study revealed substantial alteration in gene expression profiles over the period of 30 days. This is consistent with previous studies, which utilized next-generation sequencing approaches to understand adipogenesis [19,22,38,39,40]. However, most of these studies use mature sources of adipogenesis, whereas this study utilizes an earlier phase of cell commitment and specificity, thereby highlighting the benefit of studying adipocyte differentiation from ESCs. This study demonstrated the expression of over 15,000 genes that were involved in transcriptional regulation, cell cycle modulation, and DNA repair. Furthermore, the enrichment of GO terms related to lipid metabolism and fat cell differentiation further supports the specificity and relevance of our differentiation model.

Using, specifically, GO term GO:0045444~fat cell differentiation for RRA, we identified key regulatory genes central to adipogenesis in ESCs. Genes, such as PPARG, CEBPA, and EP300, emerged as central players in protein–protein interaction networks, underscoring their pivotal roles in adipocyte differentiation. Moreover, gene candidates, like *Atf5, Ccnd1*, and *Nr4a1*, highlighted by their consistent appearance in top-ranked gene lists across datasets, suggest potential new targets for further investigation into adipogenic regulation. These genes were further investigated for displaying distinctive expressions throughout the differentiation period. More specifically, *Atf5*, *Ccnd1*, and *Nra41* RT-PCR expressions were shown to correspond with the RNA-Seq data.

Studies have shown the roles of these genes and other early regulators (identified in the RNA-Seq data) in adipogenesis. These were observed to be uniquely differentially expressed in the non-differentiating conditions (D30(−)). For instance, *Klf4*, *Jdp2*, and *Smad6* are upregulated early in the differentiation process. *Klf4*, a member of the Kruppel-like factor family, is well-known for its role in maintaining pluripotency and has been shown to repress adipogenesis in preadipocytes by inhibiting the expression of adipogenic markers, such as PPARγ [41,42]. However, its role during the early stages of ESC differentiation into adipocytes suggests a dual function, potentially acting as a gatekeeper that balances self-renewal and lineage commitment. Similarly, *Jdp2*, a member of the AP-1 family, is known to interact with C/EBPβ, a critical transcription factor in adipogenesis [43,44]. Jdp2 facilitates adipocyte differentiation by modulating the activity of C/EBPβ, highlighting its importance in the transcriptional regulation of adipogenic genes. Interestingly, *Jdp2* has also been shown to directly regulate activating transcription factor (ATF) proteins, specifically ATF4 [45]. Furthermore, Smad6, an inhibitory Smad, plays a role in the negative regulation of BMP signaling, which is crucial for adipogenesis [46]. The upregulation of Smad6 early in differentiation might indicate a fine-tuning mechanism that controls the extent of BMP signaling, thereby influencing the commitment to the adipogenic lineage.

Further to these genes, there were also genes that were associated as mediators of metabolic and cell cycle regulation. Among these, *Ccnd1* was shown to be downregulated in both the data and, as mentioned previously, in RT-PCR. *Ccnd1*, typically associated with cell cycle regulation, has been shown to inhibit adipogenesis by blocking the activity of PPARγ and C/EBPα, two master regulators of adipocyte differentiation [47]. *Ccnd1* downregulation has been observed to be necessary for the progression of adipogenesis [47,48]. Therefore, the observed downregulation of *Ccnd1* in the current study in the later stages of differentiation might be necessary for the full activation of these adipogenic pathways, underscoring its role in the temporal regulation of cell fate decisions. Another mediator identified included *Ffar4*, which is a receptor involved in fatty acid sensing that has been implicated in promoting adipogenesis through the enhancement of insulin sensitivity and anti-inflammatory effects [49,50].

Further to the validation of *Ccnd1* in this study, *Atf5* and *Nr4a1* have also been shown to be involved in adipocyte differentiation. *Atf5*, a member of the ATF/CREB family, has been implicated in the regulation of adipogenesis through its interaction with C/EBPβ [51]. This study found ATF5 expression to be associated with the obesity-related phenotype, suggesting its role in promoting adipocyte differentiation. On the other hand, *Nr4a1*, which is an orphan nuclear receptor, is involved in the regulation of metabolic processes and has been shown to influence adipocyte maturation and lipid metabolism [52,53]. The consistent upregulation of *Nr4a1* during differentiation, in the current study, indicates its potential role in fine-tuning the adipogenic program, possibly through interactions with other nuclear receptors or transcription factors. Another factor, which was observed in this study to be highly ranked, was the dynamic expression of EP300. EP300, a histone acetyltransferase, is known to coactivate PPARγ and C/EBPα, thereby enhancing the transcription of adipogenic genes [54,55]. Its central role in the protein–protein interaction network identified in our study further supports its importance in the regulation of adipogenesis. Interestingly, this study also identified *Angptl8* (Gm6484) to be uniquely differentially expressed in differentiating conditions ((+)/D0). *Angptl8*, also known as betatrophin, is a liver-derived protein, which has been implicated in lipid metabolism and adipocyte differentiation [56]. Studies have shown that Angptl8 plays a role in promoting lipid accumulation in adipocytes and modulating systemic lipid metabolism, making it a potential target for understanding fat cell differentiation and metabolic regulation [57,58,59].

Although this study leverages the robustness of RRA to provide a comprehensive dynamical view of the transcriptome, especially during adipogenesis in ESCs, it is still limited. This is mainly due to having key adipogenic factors, such as (but not limited to) *Cebpa*, *Cebpb*, *Cebpd¸* and *Pparg,* already being expressed at a plateau level. This leads to the inability to observe distinctive changes in expression between the differentiating and non-differentiating conditions and might suggest that this distinctive expression occurs at an earlier timepoint.

The use of RRA in this study was particularly significant from a biological perspective. RRA is a powerful computational tool that excels in integrating data from multiple sources or experimental conditions, even in the presence of noise and variability [33]. This approach allows for the identification of key regulatory genes that may be subtle or obscured in traditional differential expression analysis [33]. By ranking genes based on their expression changes across various conditions, RRA helps to prioritize biologically relevant candidates that are consistently associated with the adipogenic process [60], providing a more reliable identification of genes that are central to cellular differentiation. This current work is the first to utilize this approach using mouse ESCs as a model for adipogenesis.

The significance of RRA lies in its ability to consolidate complex transcriptomic data into meaningful biological insights [61]. In the context of adipogenesis, this approach enabled the identification of genes that are not only differentially expressed but also functionally relevant to the adipogenic phenotype. For instance, RRA highlighted the roles of EP300, Atf5, and Nr4a1, which may not have been as prominent in a traditional analysis due to the complex and dynamic nature of gene expression during differentiation. However, the limitations observed in our study underscore the importance of considering earlier time points in future experimental designs. Investigating earlier stages of differentiation could reveal significant changes in gene expression that are critical for initiating adipogenesis. Additionally, linking these early transcriptional changes to secreted protein functionalities would provide further insights into the molecular mechanisms driving adipocyte differentiation.

Despite these limitations, the findings from this study contribute significantly to basic biology and offer potential applications in clinical settings, particularly in the context of metabolic diseases. The insights gained by RRA pave the way for future research aimed at enhancing therapeutic strategies and deepening our understanding of adipose tissue biology. By identifying key regulatory networks involved in adipogenesis, this study offers a foundation for developing targeted interventions that could modulate adipocyte function and potentially treat conditions like obesity and insulin resistance.

## 4. Materials and Methods

### 4.1. mESC Culture and Adipocyte Differentiation

Mouse ESC (mESC::*hb9*::GFP) of the strain HBG3 [37] was used throughout this study. Cells were grown and maintained under ES growth media (Knockout™ DMEM (Thermo Fisher Scientific, Carlsbad, CA, USA), 14% Fetal Bovine Serum (Corning^®^, Woodland, CA, USA), 1× 2-Mercaptoethanol (Thermo Fisher Scientific, Carlsbad, CA, USA), 3× Mix [1× L-Glutamine (Thermo Fisher Scientific, Carlsbad, CA, USA), 1× NEAA (Thermo Fisher Scientific, Carlsbad, CA, USA), and 1× Nucleosides (Thermo Fisher Scientific, Carlsbad, CA, USA)], and 10^7^ U/mL Leukemia inhibitory factor [LIF] (Thermo Fisher Scientific, Carlsbad, CA, USA)) under humidified conditions of 5%CO_2_/37 °C. Differentiation media consisted of the same components as ES growth media but without the addition of LIF. Differentiation was carried out first through the formation of embryoid bodies (EBs) through the “hanging drop” method with a cellular concentration of 1 × 10^5^ cells/mL for 2 days [18,19,20]. EBs were collected and plated into 0.1% gelatin-coated wells followed by treatment with 1 µM of Retinoic Acid and 12.5 µg/mL of Ascorbic Acid in differentiation media the following day for 3 days. From day 7, attached EBs were treated with differentiation media supplemented with 12.5 µg/mL of Ascorbic Acid, 3 nM of Thyroid T3, and 0.5 µg/mL of insulin. These media were changed every 2 to 3 days up until day 25. On day 25, differentiation media were removed, and serum-free media were added for a period of 5 days (day 30). Cells were collected for further analysis on day 30.

### 4.2. Total RNA Extraction and cDNA Synthesis

Total RNA was extracted from all mouse ESC conditions using two methods when needed. For RNA-Seq libraries’ preparation, a combination of TriZol (Life Technologies™, Thermo Fisher Scientific, Carlsbad, CA, USA) [62] and the RNeasy Mini Kit (QIAGEN, Hilden, Germany, Cat.no. 74104) was used, with modifications from the manufacturer’s protocols. Cells in each well were washed once with 1 mL of PBS prior to the addition of 1 mL of TriZol reagent. TriZol lysates were added to fresh 1.5 mL tubes. Approximately 0.2 mL of chloroform was added per ml of TriZol and centrifuged at a speed of 12,000 rpm at 4 °C for 15 min. The upper aqueous phase (~400 mL in volume) was transferred to a fresh 1.5 mL tube. An equivalent volume of 70% ethanol was mixed with the RNA solution, and downstream purification was performed using an RNeasy Mini Spin Column (QIAGEN, Hilden, Germany, Cat.no. 74104) according to the manufacturer’s protocol [63]. The second method was followed accordingly and solely with TriZol manufacturer instructions [62]. RNA concentrations were determined using a Thermo Scientific™ NanoDrop 2000™ (Thermo Fisher Scientific, Carlsbad, CA, USA), whereas the integrity of RNA (RIN) was evaluated on a Tape station 4200 (Agilent Technologies, Inc., Santa Clara, CA, USA) RNA Screen Tape. RIN numbers of more than 8 were further used for library preparation.

### 4.3. cDNA Synthesis and RT-PCR

For complementary DNA (cDNA) synthesis, a concentration amount of (0.1 ng) total RNA, from either method, was used as a template using a First-Strand Synthesis Kit (Invitrogen™, Carlsbad, CA, USA) according to the manufacturer’s instructions. cDNA synthesized was further diluted 1:50 in ddH2O prior to its use as a template for real-time PCR (RT-PCR) using SYBR Green (Thermo Scientific™). A total volume of 10 µL was prepared for the SYBR Green qPCR reaction [2.5 µL diluted cDNA product or nH2O, 2.5 µL 2 mM forward + reverse primer mix (Integrated DNA Technologies, Inc., Coralville, IA, USA) and 5 uL of SYBR Green]. The qPCR thermal cycle utilized an Applied Biosystems™ StepOnePlus™ platform (Applied Biosystems™, Waltham, MA, USA), with one cycle of 50 °C for 2 min and 95 °C for 10 min followed by 45 cycles of 95 °C for 15 s and 60 °C for 1 min. The expression of gene targets’ values was normalized to housekeeping gene β-actin and estimated using the ΔΔCt approach. Primers included the genes *Pparg*, *Cebpb*, *Fabp4*, *Oct4*, *Ccnd1*, *Atf5*, and *Nr4a1* (Supplementary File: Table S5). The housekeeping gene *Bact* was used as reference control.

### 4.4. Oil Red O Staining and Quantification

Mouse ESCs, under differentiating and non-differentiating conditions, were washed with 1 mL of 1× PBS (Corning^®^, Woodland, CA, USA) once before fixing for 5 min with 4% paraformaldehyde in PBS at room temperature. Thereafter, cells were washed three times with 1 mL of PBS and once with 60% isopropanol and completely air-dried. ESCs were then stained with Oil Red O (ORO) [6 ORO:4 ddH2O ratio] (Sigma©, Saint Louis, MO, USA) for 10 min at room temperature. Excess stain residue was removed with four ddH2O washes. Approximately 1 mL of PBS was then added for microscopic visualization. Images were processed at 460× magnification using a 20× objective lens, with transmitted bright-field light using EVOS^®^ FLoid^®^ Cell Imaging Station (Thermo Fisher Scientific, Carlsbad, CA, USA). For quantification, ORO stain particles were eluted with 100% isopropanol and analysed using Thermo Scientific™ Varioskan^®^ Flash (Thermo Fisher Scientific, Carlsbad, CA, USA) for spectrophotometry readings at 500 nm.

### 4.5. RNA-Sequencing Library Preparation and Sequencing

Libraries were prepared using the NEBNext Ultra II RNA Library Prep Kit (New England Biolabs®, Ipswich, MA, USA) from New England Biolabs following the manufacturer’s recommendations. Total RNA samples (500 ng) were subjected to cDNA construction for Illumina sequencing, in accordance with the protocol for the mRNA-Seq sample preparation kit. Oligo(dT) (Illumina, Inc., San Diego, CA, USA) magnetic beads were used to isolate poly(A) RNA from the total RNA samples. The mRNA was fragmented, and the first-strand cDNA was reverse-transcribed using random primers, following second-strand cDNA synthesis. The resulting double-stranded cDNA was ligated to NEBNext^®^ adaptors after being end-repaired and A-tailed. Finally, PCR amplification of 12 cycles was performed for enrichment, producing a 350–400 bp fragment including adapters. The fragment size and purity of the libraries were assessed on a Tape station 4200 (Agilent Technologies, Inc., Santa Clara, CA, USA) using high-sensitivity D5000 Screen Tape and quantified with the Qubit 4.0 Fluorometer (Thermo Fisher, Waltham, MA, USA). The quantifications of the libraries required for RNA-seq were determined through real-time qPCR using a KAPA library quantification kit for the Illumina platform (KAPABIOSYSTEMS, Cape Town, South Africa). Enriched cDNA libraries were sequenced using the Illumina NextSeq 500/550 system with High Output v2 kit (150 cycles, PE), (Illumina, Inc., San Diego, CA, USA).

### 4.6. Computational Analysis

Correlation analyses, including principal component analysis (PCA) and distance heatmap dendrogram analysis, were generated using RNA-Seq NASCAR 2.0 via the New York University Abu Dhabi Centre of Genomic and Systems Biology (NYUAD-CGSB) Bioinformatics Online Analysis and Visualization Portal (http://nasqar2.abudhabi.nyu.edu/) [64].

DEG lists based on comparative analysis (i.e., statistical significance [*p*-value < 0.05]) were identified via Python scripting and were used for further gene ontology (GO) enrichment analysis using the g:Profiler database (https://biit.cs.ut.ee/gprofiler/gost, accessed on 1 March 2024) [65].

### 4.7. Statistical Analysis

Statistical analysis was conducting using Student’s *t*-tests in Microsoft Excel™ (Microsoft^®^, Version 2406). Results are represented as means of at least three independent experiments, and a *p*-value < 0.05 was considered significant.

## Data Availability

RNA-Seq data associated with this study were securely saved under BioProject accession number reference PRJNA1126392 in the National Library of Medicine at the National Center of Biotechnology information (NCBI).

## Supplementary Materials

The following supporting information can be downloaded at: https://www.mdpi.com/article/10.3390/ijms25179154/s1.
