# Supplementary material for: Transcriptional Dynamics and Key Regulators of Adipogenesis in Mouse Embryonic Stem Cells: Insights from Robust Rank Aggregation Analysis
Source: Int J Mol Sci. 2024 Aug 23;25(17):9154. doi: 10.3390/ijms25179154 (PMC11395306; doi:10.3390/ijms25179154)
Supplement: Supplementary file 1 [file ijms-25-09154-s001.zip › ijms-3103840-supplementary.pdf]

Table S1

**Term**	**Count**	**PValue**
GO:0045944~positive regulation of transcription from RNA polymerase II promoter	941	8.14E-38
GO:0006397~mRNA processing	320	4.55E-31
GO:0007049~cell cycle	512	2.24E-30
GO:0006974~cellular response to DNA damage stimulus	424	2.42E-26
GO:0000122~negative regulation of transcription from RNA polymerase II promoter	765	7.10E-26
GO:0007275~multicellular organism development	799	1.57E-25
GO:0006357~regulation of transcription from RNA polymerase II promoter	1043	8.37E-25
GO:0045893~positive regulation of transcription, DNA-templated	552	1.75E-24
GO:0006281~DNA repair	319	3.11E-22
GO:0006915~apoptotic process	492	4.45E-22

Table S2

Term	Count	PValue
GO:0045944~positive regulation of transcription from RNA polymerase II promoter	955	6.58E-39
GO:0007049~cell cycle	525	6.64E-34
GO:0006974~cellular response to DNA damage stimulus	436	5.73E-30
GO:0000122~negative regulation of transcription from RNA polymerase II promoter	779	2.19E-27
GO:0006397~mRNA processing	313	3.16E-25
GO:0045893~positive regulation of transcription, DNA-templated	560	4.06E-25
GO:0006915~apoptotic process	501	2.40E-23
GO:0016310~phosphorylation	524	4.45E-23
GO:0006357~regulation of transcription from RNA polymerase II promoter	1048	9.71E-23
GO:0007275~multicellular organism development	799	1.04E-22

Table S3

Term	Count	PValue
GO:0006629~lipid metabolic process	517	1.08E-11
GO:0045444~fat cell differentiation	80	1.31E-05
GO:0006631~fatty acid metabolic process	145	7.41E-05
GO:0008654~phospholipid biosynthetic process	52	8.89E-05
GO:0015012~heparan sulfate proteoglycan biosynthetic process	27	0.000209
GO:0045599~negative regulation of fat cell differentiation	45	0.000449
GO:0019216~regulation of lipid metabolic process	36	0.000963
GO:0030206~chondroitin sulfate biosynthetic process	19	0.002043
GO:0001676~long-chain fatty acid metabolic process	26	0.002854
GO:0050873~brown fat cell differentiation	35	0.002909
GO:0019915~lipid storage	27	0.004659
GO:0006635~fatty acid beta-oxidation	44	0.007443
GO:0045600~positive regulation of fat cell differentiation	42	0.007586
GO:0008610~lipid biosynthetic process	22	0.01245
GO:0033539~fatty acid beta-oxidation using acyl-CoA dehydrogenase	12	0.013666
GO:0006869~lipid transport	118	0.014802
GO:0050872~white fat cell differentiation	21	0.017689
GO:0060612~adipose tissue development	38	0.021681
GO:0006633~fatty acid biosynthetic process	52	0.02335
GO:0009395~phospholipid catabolic process	20	0.024938
GO:0006644~phospholipid metabolic process	37	0.027785
GO:0006665~sphingolipid metabolic process	33	0.047208
GO:0000038~very long-chain fatty acid metabolic process	18	0.04829

Table S4

Term	Count	PValue
GO:0006629~lipid metabolic process	534	2.82E-14
GO:0045599~negative regulation of fat cell differentiation	45	0.000733
GO:0006635~fatty acid beta-oxidation	47	0.000796
GO:0045444~fat cell differentiation	76	0.000853
GO:0006631~fatty acid metabolic process	142	0.001062
GO:0008654~phospholipid biosynthetic process	49	0.002941
GO:0008610~lipid biosynthetic process	23	0.00471
GO:0006644~phospholipid metabolic process	39	0.008453
GO:0019216~regulation of lipid metabolic process	34	0.010829
GO:0045165~cell fate commitment	60	0.01394
GO:0055088~lipid homeostasis	43	0.014144
GO:0048665~neuron fate specification	22	0.015912
GO:0019915~lipid storage	26	0.017947
GO:0016042~lipid catabolic process	85	0.019041
GO:0045600~positive regulation of fat cell differentiation	41	0.022944
GO:0034389~lipid particle organization	16	0.026081
GO:0060612~adipose tissue development	38	0.029833
GO:0090336~positive regulation of brown fat cell differentiation	15	0.037659
GO:0015908~fatty acid transport	25	0.043427

Table S5

**Primer**	**Forward**	**Reverse**
Cebpb	5’-ATCGACTTCAGCCCCTACCT-3’	5’-TAGTCGTCGGCGAAGAGG-3’
Fabp4	5’-GGATGGAAAGTCGACCACAA-3’	5’-TGGAAGTCACGCCTTTCATA-3’
Pparg	5’-AAGACAACGGACAAATCACCA-3’	5’-GGGGGTGATATGTTTGAACTTG-3’
Oct4(Pou5f1)	5’-TTGCAGCTCAGCCTTAAGAAC-3’	5’-TCATTGTTGTCGGCTTCCT-3’
Nr4a1	5'-TTGAGTTCGGCAAGCCTACC-3'	5'-GTGTACCCGTCCATGAAGGTG-3'
Atf5	5'TGGGCTGGCTCGTAGACTAT-3'	5'-GTCATCCAATCAGAGAAGCCG-3'
Ccnd1	5'-GCGTACCCTGACACCAATCTC-3'	5'-CTCCTCTTCGCACTTCTGCTC-3'
Bact	5'-CATTGCTGACAGGATGC-3'	5'-TGCTGGAAGGTGGACA-3'
